# An Observational Cohort Study and Nested Randomized Controlled Trial on Nutrition and Growth Outcomes in Moderate and Late Preterm Infants (FLAMINGO)

**DOI:** 10.3389/fnut.2021.561419

**Published:** 2021-03-08

**Authors:** Andreas Kakaroukas, Marieke Abrahamse-Berkeveld, Janet E. Berrington, Richard J. Q. McNally, Christopher J. Stewart, Nicholas D. Embleton, Ruurd M. van Elburg

**Affiliations:** ^1^Newcastle Neonatal Service, Royal Victoria Infirmary, Newcastle upon Tyne Hospitals National Health Service (NHS) Foundation Trust, Newcastle upon Tyne, United Kingdom; ^2^Danone Nutricia Research, Utrecht, Netherlands; ^3^Translational and Clinical Research Institute, Newcastle University, Newcastle upon Tyne, United Kingdom; ^4^Faculty of Medical Sciences, Population and Health Sciences Institute, Newcastle University, Newcastle upon Tyne, United Kingdom; ^5^Emma Children's Hospital, Amsterdam University Medical Centers (Amsterdam UMC) Amsterdam, Amsterdam, Netherlands

**Keywords:** prematurity, late preterm, moderate preterm, growth, nutrition, breastfeeding, lipid, body composition

## Abstract

**Background:** Over the past decades, the preterm birth rate has increased, mostly due to a rise in late and moderate preterm (LMPT, 32–36 weeks gestation) births. LMPT birth affects 6–7% of all births in the United Kingdom and is associated with increased morbidity risk after birth in infancy as well as in adulthood. Early life nutrition has a critical role in determining infant growth and development, but there are limited data specifically addressing LMPT infants, which was the rationale for the design of the current study.

**Objective:** The Feeding Late and Moderate Infants and Growth Outcomes (FLAMINGO) study aims to improve understanding of the longitudinal growth, nutritional needs, and body composition of LMPT infants as well as their microbiome development and neurodevelopment. In addition, having a nested non-inferiority trial enables evaluation of the nutritional adequacy of a concept IMF with large milk phospholipid-coated lipid droplets comprising dairy and vegetable lipids. The primary outcome of this RCT is daily weight gain until 3 months corrected age.

**Methods:** A total of 250 healthy LMPT infants (32+0–36+6 weeks gestational age) with birth weight 1.25–3.0 kg will be recruited to the cohort, of which 140 infants are anticipated to be enrolled in the RCT. During six visits over the first 2 years of life, anthropometry, body composition (using dual energy X-Ray absorptiometry), feeding behavior, and developmental outcomes will be measured. Saliva and stool samples will be collected for oral and gut microbiota assessment.

**Discussion:** The FLAMINGO study will improve understanding of the longitudinal growth, body composition development, and feeding characteristics of LMPT infants and gain insights into their microbiome and neurodevelopment.

**Study Registration:** www.isrctn.com; Identifier ISRCTN15469594.

## Introduction

Worldwide, the rate of preterm birth (<37 weeks gestation) is increasing (1, 2), and this represents an important burden on health care systems (3). In some countries, rates of preterm birth exceed 10–15%, but in all countries, the major contributors are births between 32 and 36 weeks' gestation (1), so-called late and moderately preterm infants (LMPT). Compared with full-term born infants, LMPT infants are more likely to require feeding support and specific medical management after birth as well as having increased risks of infections, hypoglycemia, jaundice, and delayed breastfeeding along with a range of other feeding issues (4–6). In later infancy, LMPT infants have higher rates of poor growth, feeding difficulties, and behavioral problems compared with those born at full term, and there are also data to show higher rates of adverse metabolic programming in adulthood (4, 5, 7–9).

Breastfeeding results in better outcomes during infancy as well as throughout the life course for infants born both term and preterm. Recent studies in LMPT infants show important associations between breastfeeding at discharge and a range of later feeding and behavioral issues and strongly suggest that greater efforts must be made to support and enhance breast milk exposure in these infants (10–13). Initiation and successful breastfeeding rates in LMPT infants are still lower than in infants born full term (6); therefore, many LMPT infants also receive formula milk in addition to breastfeeding or are exclusively formula fed. However, there are no detailed studies of breastfeeding success (i.e., duration of any or exclusive breastfeeding) in the first few weeks and months in LMPT infants. Studies are, therefore, needed that determine factors that impact breastfeeding success, including maternal factors, such as pre-existing morbidities (e.g., diabetes), pregnancy-associated medical illness (e.g., hypertension, placental dysfunction), labor and delivery factors (e.g., reason for preterm delivery, mode of delivery, etc.), demographic factors (e.g., maternal age, socioeconomic status etc.), and infant factors, such as admission to the NICU, length of hospital stay, and requirement for additional nutrition or feeding support. A better understanding of such factors associated with breastfeeding success informs the design of prospective trials that aim to improve longer-term infant and child outcomes. Despite the many studies exploring nutritional requirements, growth, body composition, and numerous other health outcomes in VPT infants, there are virtually no prospective trials and few observational studies in those born LMPT. The role of nutrient enrichment or supplementation that is widely promoted for VPT infants to improve cognitive outcomes is uncertain in those born LMPT (14). Moreover, rapid growth promotion and/or catch-up in weight in early life might adversely affect later metabolic outcomes (15, 16), and the balance of risks and benefits for cognitive and metabolic outcomes might differ substantially between VPT and LMPT infants. Limited studies suggest that formula-fed LMPT infants achieve higher fat mass and higher fat mass percentage at 36 weeks compared with breastfed infants (14), which might relate to their observed higher risks for obesity and metabolic problems in adulthood (5, 16). Importantly, there are no consistent controlled trial data to show a cognitive benefit of nutrient enrichment in the post-discharge period in otherwise well LMPT infants. Current practice in our unit is to use a standard term formula in which breast milk is not available even for LMPT infants weighing <2.5 kg at birth although feeding practices mean that most LMPT infants may take several days to achieve full enteral feeds and, therefore, meet estimated nutrient intake recommendations. However, there is substantial variation in feeding practices for LMPT infants, reflecting the lack of evidence on which to base nutritional recommendations (17, 18). This variation in current nutritional practices in part reflects uncertainty around how best to establish good linear growth that might support more appropriate lean mass accretion and brain growth against the potential metabolic and cardiovascular longer-term risks associated with excessive infant weight gain (9, 19). A better understanding of feeding practices and their relation to growth, body composition, and brain development in LMPT infants is, therefore, of key importance to develop evidence-based nutritional guidelines for this high-risk population.

When mothers are unable to or choose not to breastfeed, there remain important uncertainties around optimal formula milk composition, and this is especially important in higher risk populations, such as those born LMPT. Breast milk substitutes should aim to provide nutritional and functional properties as close as possible to those of human milk, reflecting appropriate levels of macronutrients and micronutrients as well as functional ingredients to optimally support growth and development of infants.

Lipids are a key constituent of all mammalian milks, and there are important variations in quantity and quality between human and cow milk–based infant formula. Lipids are the main energy source for the infant. In breast milk, lipids are mostly present as triacylglycerols (20) in the core of large lipid droplets enveloped by a three-layered membrane mainly consisting of phospholipids, membrane-specific proteins, and cholesterol (20–22). Compared with breast milk, standard infant formula shows distinct differences in overall fatty acid composition (23) and the structure of triglycerides (24) as well as the physical properties of its lipid droplets, i.e., being smaller in size and having proteins as a main emulsifier (lacking a membrane) (20–22), which are likely to affect lipid digestion and metabolism in infants (25–27). A concept infant milk formula (IMF) has been developed with physical properties of lipid droplets closer to those in human milk (28) (i.e., large lipid droplets with milk phospholipids adhering to the interface and containing a mixture of dairy and vegetable lipids). In a proof-of-concept study in adult men aimed to evaluate the metabolic effects of the concept IF, earlier peak concentrations of glucose and insulin were observed after consuming the concept IF, and postprandial triacylglycerol concentrations tended to increase faster compared with consuming a standard IF (29). In murine nutritional programming models, this concept IMF was shown to prevent excessive adiposity and adverse metabolic outcomes (30–32) as well as improved specific cognitive behaviors (33). Hence, it is postulated that this concept IMF may bring the physiological properties of IMF closer to those of human milk. In a recent randomized double-blind equivalence trial, this concept IMF supported adequate growth, was well-tolerated, and safe for use in healthy term infants (34). Given the indications for suboptimal metabolic and cognitive development with LMPT birth, it is postulated that this concept IMF could support adequate growth and development in LMPT infants when breast milk is not available.

The aim of the FLAMINGO cohort study is to collect data and improve understanding on the longitudinal growth, body composition, and feeding characteristics as well as their microbiome and cognitive development. In addition, the aim of the nested non-inferiority RCT is to evaluate the nutritional adequacy of a concept IMF, including weight gain primarily and other anthropometric parameters and gastrointestinal characteristics in comparison to a control commercial formula.

## Materials and Methods

### Study Aims and Objectives

Despite the fact that LMPT infants have higher rates of short- and longer-term morbidities, there is no strong evidence to guide nutritional management if mothers do not breastfeed or are unable to provide a sufficient volume of breast milk. In addition, breastfeeding rates in LMPT infants are frequently lower than in term-born infants, and a better understanding of why this occurs may enable improved support. A greater understanding of the feeding characteristics and growth of this population can also provide background knowledge to support nutritional recommendations. This study aims to (1) assess feeding characteristics and growth patterns in LMPT infants during the first 2 years using an observational, prospective cohort study design and (2) evaluate growth of infants, defined as daily weight gain until 3 months-corrected age, fed with a concept IMF comprising large, milk phospholipid-coated lipid droplets containing a mixture of dairy and vegetable lipids in a randomized, controlled, double-blind, and non-inferiority trial nested within the cohort.

### Setting

The FLAMINGO study will be conducted at the Royal Victoria Infirmary (RVI) in Newcastle upon Tyne, in accordance with good clinical practice. The study was reviewed by the North East–York Research Ethics Committee and received approval by the NHS Health Research Authority (IRAS project ID: 237542) and is registered at ISRCTN (ISRCTN15469594).

In addition, eligible infants for the intervention study who are born in other nearby hospitals will be referred to the lead site for consideration of enrollment. These infants should live within a reasonable traveling distance to the leading site, determined after discussion with health care teams and parents to be a radius of ~12 miles. Local teams will identify formula-fed infants who are eligible for the RCT and ask families' consent to be referred to the lead site where the trial will be discussed in detail and enrolled after parental consent.

### Subjects and Study Design

Eligible infants are medically stable breast, formula, or mixed milk fed meeting the following inclusion criteria: (a) born between 32+0–36+6 weeks gestation, (b) birth weight between 1.25 and 3.0 kg, (c) enrolled in the study before they reach 4 weeks after the term equivalent age (TEA), and (d) written informed consent. Following low recruitment rates to the RCT, the initial criteria of a birth weight between 1.25 and 2.5 kg and an enrollment window prior to TEA were adapted after 9 months of the study starting to increase the upper limit for birth weight from <2.5 to <3 kg and an enrollment age increased from prior to term to prior to 4 weeks after TEA. We exclude infants who have congenital or medical problems likely to affect growth, by which there are significant child protection concerns or parental substance misuse. Infants already participating in other interventional studies involving investigational or marketed products likely to impact growth and nutrition are not eligible nor are cases in which parents are unable to comply with the study procedures.

This prospective cohort study was designed with careful consideration of the UNICEF Baby Friendly Initiative (35), aiming to fully support maternal wishes to breastfeed. For this purpose, we designed a 2-step enrollment for participation in the RCT. Parents of all eligible LMPT infants are invited initially to join the FLAMINGO cohort, and there is no discussion of the RCT element. Subsequently, and only if parents independently disclose that their intention is to formula feed their baby and when the infant is solely or pre-dominantly formula fed (no more than one breast feed per day offered) before 4 weeks corrected age, the possibility to take part in the RCT is discussed. In addition to the cohort inclusion criteria, the RCT inclusion criteria includes (a) age at randomization <4 weeks after TEA, (b) pre-dominantly formula feeding (no more than 1 breastfeed a day), and (c) additional second written consent by the parents or legal guardian. Infants who require a special diet other than a standard cow's milk formula are not eligible. This 2-step enrollment approach was developed in conjunction with infant feeding support staff and parents. Breastfed infants will serve as a reference group for the RCT intervention groups. Only those infants whose mother is still fully breastfeeding at TEA and meeting all other inclusion criteria are included in this reference group. No complementary feeding is recommended before 3 months corrected age in any of the feeding groups.

Following signed informed consent for the intervention study, the infant is randomized using an online platform (www.sealedenvelope.com) and assigned to either a standard term formula milk (control) or the intervention formula. Both formulae have similar taste and nutrient concentration with the only difference being the fat globule structure as previously described. The formula milk is coded using letters A, B, C, or D with two letters for each type of milk, meaning that the research team and families are blinded to study group allocation. Parents are provided with the formula and are asked to use it until 6 months corrected age. Stratification will be applied for multiple births (multiple or singleton) and for gestation (32–33 or 34–36 weeks).

In total, five different feeding groups are expected to become apparent in this FLAMINGO cohort study: a breastfeeding reference group, a group of predominantly formula-fed infants whose parents declined participation in the RCT, a group of mixed-fed (combining formula and breast feeding at TEA) infants who were not eligible for the RCT, and the two groups of formula-fed infants randomized to receive one of the intervention formulas.

### Products Used in FLAMINGO

The intervention formulas are isocaloric (66 kcal/100 ml); contained similar amounts of protein (1.3 g/100 mL), lipids (3.4 g/100 mL), and scGOS/lcFOS pre-biotic mixture (9:1, 0.8 g/100 mL); and were manufactured per good manufacturing practices (ISO 22000) and compliant with Directive 2006/141/EC on the composition of infant formulas.

The key differences between the study IMFs were the following: (1) the size of their lipid droplets, (2) the coating of their lipid droplets, and (3) the origin of their lipid sources. The control IMF is a vegetable oil–based standard IMF containing lipid droplets with a volume-based mode diameter of 0.5 μm and proteins as main emulsifiers. The concept IMF (Nuturis, patent EP2825062A1; Nutricia Research) contains a mixture of vegetable (52%) and dairy lipids (48%), including milk phospholipids, introducing a 3-fold increase of sn-2 palmitic acid compared with the control IMF (36% compared with 12% of total palmitic acid). The lipid droplets in the concept IMF have a volume-based mode diameter of 3–5 μm and an interface pre-dominantly composed of milk phospholipids following an adapted production process (28).

### Supplementation

The FLAMINGO population includes infants who are low birth weight (<3 kg) and LMPT. Although these babies have increased needs for iron and vitamin D, in many hospitals, LMPT infants do not receive routine follow-up and may not receive advice on supplementation following discharge. The international recommendations are to supplement breastfed infants with vitamin D from birth and all other infants after the age of 1 year (36, 37). The study formulae are adequately supplemented with vitamin D, but breastfed infants in the study require supplementation. This is provided as Dalivit 0.3 ml/day containing 280 IU of vitamin D or DDrops 0.5 ml/day containing 400 IU. From 6 months corrected age, all breastfed infants are recommended to continue supplementation with vitamin D using suitably available products.

The control and intervention formulae have similar concentrations of iron, 0.53 mg/100 ml and 0.52 mg/100 ml, respectively, providing ~1 mg/kg/day. To achieve the recommended intake of 2 mg/kg/day, infants in the study are treated as per current recommendations (37) and supplemented with 1 ml of ferrous federate from 2 to 3 weeks of age (equivalent to 5.5 mg of elemental iron) until ~6 months when most are receiving two solid feeds per day.

### Data Collected

#### Demographic, Clinical, and Anthropometric Data

**Figure 1:**
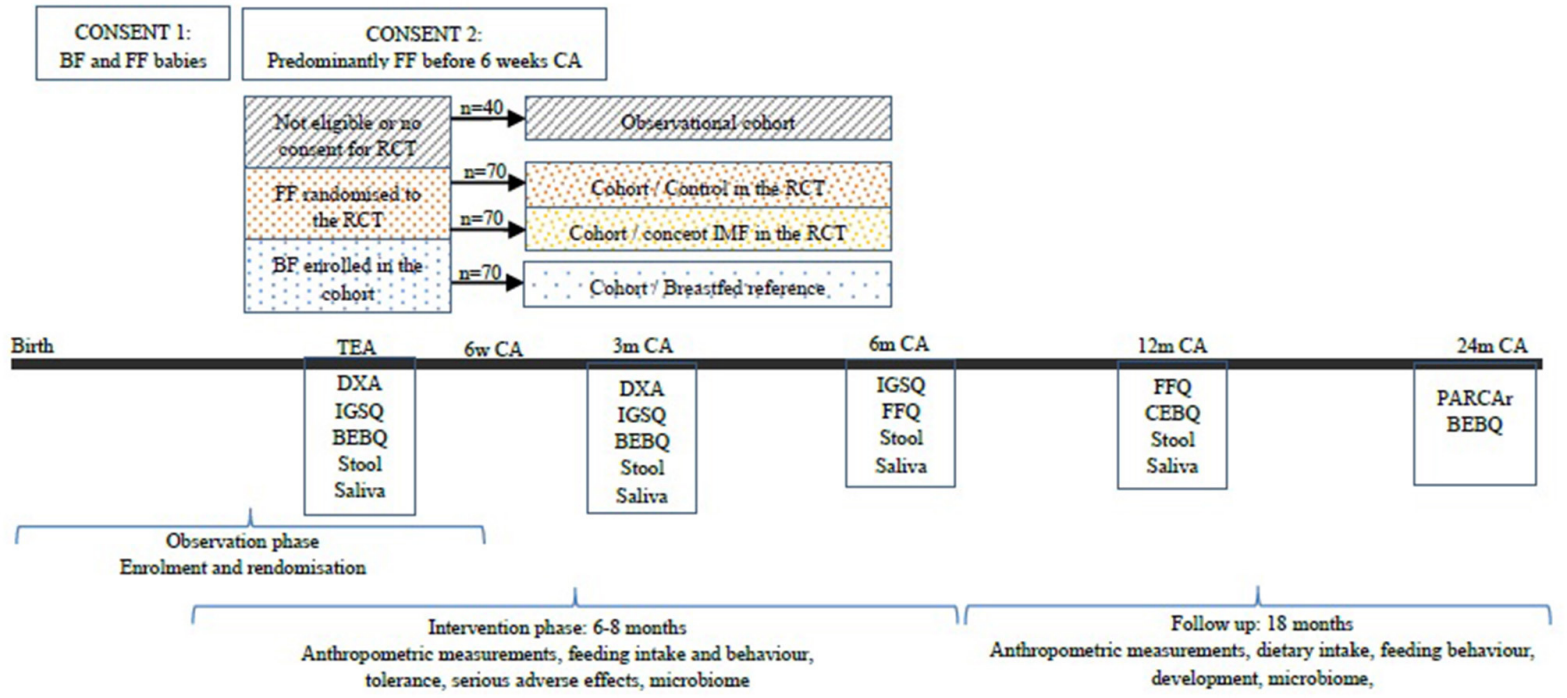
Enrolment approach and study design of the FLAMINGO intervention study. BF, breastfed; FF, formula fed; CA, corrected age; RCT, randomized controlled trial; IMF, imfant milk formula; DXA, dual energy X-Ray absorptiometry; IGSQ, infant gastrointestinal symptoms questionnaire; BEBQ, baby eating behavior questionnaire; FFQ, food frequency questionnaire; CEBQ, children eating behavior questionnaire; PARCAr, parent report of children's abilities-revised.

Clinical and growth data is collected at enrollment and at clinic visits organized at TEA at 3, 6, and 12 months and 2 years corrected age (Figure 1). Demographic and other data are extracted from the infant's medical record, measurements obtained by trained research staff, and direct parental interview of feeding practices. Figure 1 shows a summary of the enrollment, the anticipated numbers of recruits and design of the FLAMINGO intervention design, and the information collected during follow-up.

We record the infant's gestation, birth weight, gender, significant medical history (e.g., confirmed sepsis, NICU admission), medication use (antibiotics, etc.), minor malformations (e.g., inguinal hernia), parental post-code (to calculate the deprivation index) (38, 39), infant age at entry in the cohort and intervention study, age at start of the study product, and age at discharge from hospital. At the clinic visit interview, parents produced the vaccination history, use of medication, or supplements and duration of any fever episodes and duration thereof. We use the maternal notes, interviews, and measurements with the parents in the clinic to collect or record parental height; weight and BMI; medical background; pregnancy; and delivery history; maternal use of antibiotics, pre-biotics, probiotics, or long-chain polyunsaturated fatty acids (LCPUFA); and maternal smoking.

At each clinic visit, we measure the infant's weight; length or height; head circumference; mid-upper arm circumference; thigh circumference; and (from visit 2) skinfolds in four areas (biceps, triceps, subscapular, and supra-iliac). These are performed by the research staff, using the same technique as per the WHO guidance (40). Anthropometry has been shown to offer a reliable evaluation for adiposity and obesity in pediatric populations (41, 42).

#### Dual Energy X-Ray Absorptiometry (DEXA) Scan

Before TEA and at 3 months corrected age, while the infant is asleep and without the use of any sedation, body composition is assessed using DEXA scans. DEXA uses a very low radiation scan with a dose of ~0.002 msV, which is much lower the radiation of a conventional chest X-ray and has been widely used in term and preterm infants to assess body composition. We use a research grade GE Lunar iDXA with encore v12 software for analysis. A specialized pediatric module is used to produce both raw densitometry information and body composition data for analysis and trending following standardized scanning techniques. We determine fat mass (g), fat free mass (g), bone mineral density (g/cm^2^), bone mineral content (g/cm), fat free mass index (kg/m^2^), fat mass index, fat mass percentage, and fat free mass percentage (43).

#### Safety and Gastrointestinal Tolerance

Gastrointestinal tolerance is assessed by the Infant Gastrointestinal Symptoms questionnaire (IGSQ). The IGSQ is based on previous questionnaires on gastrointestinal reflux, and several studies show it has high validity, reliability, and accuracy (44). To evaluate safety of the intervention formulas for babies in the RCT, parents are also asked to report any serious adverse effects that could be related to the study products for the first 12 months and to complete a 7-day diary before every visit until 6 months corrected age. The latter records typical infant complaints, such as cramps, nappy rash, regurgitation, and vomiting, the type and frequency of stooling (watery, soft, formed, or hard), and the number of feeds and amount of formula used every day.

#### Feeding Characteristics

During each visit, we record the onset and duration of any breastfeeding, exclusive breastfeeding, exclusive formula feeding, type of formula, and feeding with the study product. We assess the child's eating behavior with the Baby Eating Behavior Questionnaire, the Children Eating Behavior Questionnaire, and a Food Frequency Questionnaire. The Baby Eating Behavior Questionnaire is used at TEA and 3 months corrected age. It is designed and validated for infants exclusively fed with milk, assessing their behavior regarding satiety, slow feeding, enjoyment, and responsiveness to food (45) and has been validated showing good feasibility and acceptable internal reliability for the latter three (46, 47). It is based on the Children Eating Behavior Questionnaire, which is used at 6 months corrected age and examines 8 scales of the child's eating behavior and has been found consistent and reliable to be used in large studies (48, 49). The Food Frequency Questionnaire is used at 6 and 12 months corrected ages and is used to estimate nutritional intake although this is acknowledged to be unreliable in breastfed infants (50, 51).

#### Neurodevelopment

The neurodevelopment of all infants is assessed at 24 months using the PARCA-R questionnaire (52). This is a parental questionnaire that has been validated against the Bayley assessment and has shown good concurrent and external validity, high sensitivity, and overall good diagnostic utility with reference values for normal development adjusted for age and gender (52–54).

#### Biological Samples

Stool samples and oral swabs are collected at all clinic visits from enrollment until 12 months corrected age. If it is not possible to collect samples during the clinic visit, parents are provided with a stool kit to collect a sample at home and return by routine post. Oral swabs are collected using sterile, polystyrene, DNA-free, and RNAse-free (PurFlock Ultra 6' sterile DNA-Free) swabs that are certified to have <25 μg of human DNA and be suitable for PCR assays and DNA testing. The samples are briefly stored at −20°C until they are collected on a weekly basis and stored at −80°C. In accordance with previous work (55), stool and oral swabs are analyzed using the V4 region of the 16S rRNA gene sequencing to determine the bacterial genera. The gut microbiota in infants has been the subject of research, playing a role in immediate and long-term health outcomes, such as atopy, infections, and neurodevelopment (56–60). Pre-maturity and medical procedures related to it, e.g., tube feeding, antibiotics, prolonged hospital stay, etc., are found to be associated with different intestinal microbiota composition in late preterm compared with term infants (61, 62), which may increase the risk for subsequent health outcomes. Any samples that are not analyzed within the study are stored in the Great North Neonatal Biobank at Newcastle University for future studies (HTA license no. 12534, Ethics approval 15/NE/0334, IRAS 161883).

### Statistics

The primary outcome of the nested intervention study is the daily weight gain (g/d) from enrollment until 3 months corrected age. Non-inferiority is demonstrated when the lower bound of calculated two-sided 90% confidence intervals (CI) is above the non-inferiority margin. We compare the above daily weight gain of the infant receiving the concept IMF (intervention) and those receiving standard formula (control) using one-sided *t*-tests.

The evaluation of non-inferiority in daily weight gain as a primary objective of the RCT is based on the paucity of evidence on body composition development and the lack of data regarding the impact of feeding interventions on growth for this population. The American Academy of Pediatrics (AAP) recommends that a clinically relevant difference regarding adequate weight gain for a term infant is 3 g/day for the first 3 months of life (63). There are no equivalent European or British recommendations. We considered the above daily weight gain for the first 3 months as relevant and assumed a SD of 6 g/day based on existing data (64).

To establish non-inferiority using a margin of −3 g/d and an SD of 6 g/d, a significance level (α) of 5%, a difference of zero, and a power of 80%, a total of 50 eligible infants are needed per arm in the trial. Assuming a dropout rate of 30%, we plan to continue to recruit infants to the cohort study until ~140 subjects have joined the RCT.

Existing data for our hospital show that, in LMPT infants, ~30% are exclusively or pre-dominantly breastfed at hospital discharge. Based on previous studies, we anticipate recruiting ~70 infants who are exclusively breastfed, another 40 infants who are mixed or formula fed and whose parents declined participation in the RCT, and enrolling 140 infants into the RCT, creating a total cohort of ~250 infants. This cohort size is assumed to be sufficient to properly evaluate feeding characteristics and growth patterns in this population of LMPT infants. Previous studies exploring growth outcomes in term and late preterm infants have shown that such size of the cohort can allow extracting conclusions (34, 65, 66).

The intervention study is a non-inferiority trial, and therefore, the analysis per protocol will be performed. We will compare the characteristic of the infants with and without sufficient data. We expect these to be similar and do not anticipate that the above analysis should introduce attrition bias.

Finally, the anthropometric measurements will be cross-validated with the DEXA findings using linear regression, aiming for a high degree of correlation with this methodology (42, 67).

## Discussion

The American Academy of Pediatrics (AAP) recommends a postnatal growth for preterm infants similar to intrauterine fetal growth. Given the typical weight loss in the 1st days of life, such an outcome would require a substantial catch-up growth, which may increase the risk of obesity and other metabolic problems in later life. In LMPT infants, there are increased risks of being underweight in early life. Santos et al. found that late preterm babies are 2.57 and 3.36 times more likely to be underweight compared with term infants at 12 and 24 months, respectively, while Gupta et al. found an odds ratio of being underweight of 4.1 at 12 months (65, 68). However, LMPT birth is associated with higher rates of adverse metabolic programming in adulthood (4, 5, 7–9), potentially originating from a suboptimal early life development (excessive weight gain). Balancing any potential benefits of achieving a postnatal growth velocity similar to fetal growth against the risks of a too rapid weight gain remains an important area of active research. Specifically, the AAP recommendation refers primarily to weight gain and may not reflect the need for a more holistic approach to growth quality, including an assessment of linear and head growth and body composition. Compared with term infants, LMPT infants have a higher risk of increased fat mass for the same weight (42, 43, 69). However, there is a lack of good quality data regarding the longitudinal changes in anthropometry in LMPT infants (70).

There is wide variation in practice regarding the nutritional management of the LMPT population (17, 19). Examples of this variation include different types of feed for LMPT infants, offering either caloric and nutritional intake similar to the term infants or higher concentration of calories and nutrients by breast milk fortifiers or enriched formulas (12, 13, 17, 19). For these reasons, we designed the study aiming to determine nutritional practices, growth, and body composition as well as cognitive development of LMPT infants. It is of paramount importance to ensure that when breastfeeding does not occur, infants are fed with the most suitable products supporting optimal growth and development. To this end, optimalization of the level and quality of macronutrients and functional ingredients in infant nutrition have been and still are focus areas in pediatric research over the past decades. Previous studies suggest that lipid quality has important effects on growth, metabolism, body composition, and cognitive outcomes (29–31, 33, 34). The nested RCT aims to collect data on the growth adequacy of infants on a standard term formula as well as a concept term formula with lipid droplet characteristics closer to the fat globules in human milk.

We acknowledge that the chosen study design has important limitations. First, as for many longitudinal studies, frequent clinic visits in otherwise healthy infants up to the age of 2 years may discourage some families from participating, especially those from economically deprived areas and/or single parents. This may also be more challenging for parents of twin/triplet babies who represent a significant proportion of LMPT births. In addition, the change of the inclusion criteria necessitated by low enrollment rates may lead to inclusion of a more heterogenous sample in a population that is heterogenous by nature.

Using a 2-step enrollment approach may bring additional challenges as parents may ask why this was not explicit at initial cohort enrollment, and it is possible that this more time-consuming approach decreases consent rates to the RCT further. However, we designed this approach carefully to ensure that the Breastfeeding Friendly Initiative would not be interrupted (35), accepting that some eligible formula-fed babies who could be invited in the intervention study are missed. It is also likely that parents of some LPMT infants who are formula fed but otherwise stable may be reluctant to change the formula for their babies. Despite these challenges and our strong support for the advantages of breastfeeding, we considered it important to conduct an RCT exploring the adapted lipid quality of formula milk understanding the potential health benefit particularly for this more vulnerable population.

Previous studies suggest that lipid quality has important effects on growth, metabolism, body composition, and cognitive outcomes (29–31, 33, 34), and there is an important obligation to ensure that when breastfeeding does not occur, infants are fed with the most suitable products. The results from the FLAMINGO study will provide detailed data on LMPT infants' anthropometry, growth, nutrition, and eating behavior for their first 2 years of life and provide additional insights into factors relating to duration of breastfeeding and breast milk exposure.

## Data Availability Statement

The original contributions presented in the study are included in the article/supplementary material, further inquiries can be directed to the corresponding author.

## Ethics Statement

The studies involving human participants were reviewed and approved by North East–York Research Ethics Committee. Written informed consent to participate in this study was provided by the participants' legal guardian/next of kin.

## Author Contributions

The study was conceived by MA-B, NDE, and RMvE. The original draft of this article was produced by AK, MA-B, NDE, and RMvE. All authors have contributed to the development of the methodology, design of the study, reviewed, and edited the manuscript.

## Conflict of Interest

MA-B is an employee of Danone. RMvE was previously an employee of Danone. NDE declares research funding from Danone Early Life Nutrition and Prolacta Biosciences US, and lecture honoraria from Nestle Nutrition Institute. The remaining authors declare that the research was conducted in the absence of any commercial or financial relationships that could be construed as a potential conflict of interest.

## References

[1] Blencowe H Cousens S Oestergaard MZ Chou D Moller AB Narwal R. National, regional, and worldwide estimates of preterm birth rates in the year 2010 with time trends since 1990 for selected countries: a systematic analysis and implications. Lancet. (2012) 379:2162–72. 10.1016/S0140-6736(12)60820-4 22682464

[2] Shapiro-Mendoza CK Lackritz EM. Epidemiology of late and moderate preterm birth. Semin Fetal Neonatal Med. (2012) 17:120–5. 10.1016/j.siny.2012.01.007 22264582 PMC4544710

[3] Lisonkova S Sabr Y Butler B Joseph KS. International comparisons of preterm birth: higher rates of late preterm birth are associated with lower rates of stillbirth and neonatal death. BJOG. (2012) 119:1630–9. 10.1111/j.1471-0528.2012.03403.x 23164112

[4] Johnson S Marlow N. Early and long-term outcome of infants born extremely preterm. Arch Dis Child. (2017) 102:97–102. 10.1136/archdischild-2015-309581 27512082

[5] Kugelman A Colin AA. Late preterm infants: near term but still in a critical developmental time period. Pediatrics. (2013) 132:741–51. 10.1542/peds.2013-1131 24062372

[6] Lapillonne A Bronsky J Campoy C Embleton N Fewtrell M Fidler Mis N. Feeding the late and moderately preterm infant: a position paper of the European society for paediatric gastroenterology, hepatology and nutrition committee on nutrition. J Pediatr Gastroenterol Nutr. (2019) 69:259–70. 10.1097/MPG.0000000000002397 31095091

[7] Raju TNK Pemberton VL Saigal S Blaisdell CJ Moxey-Mims M Buist S. Long-Term healthcare outcomes of preterm birth: an executive summary of a conference sponsored by the national institutes of health. J Pediatr. (2017) 181:309–18 e1. 10.1016/j.jpeds.2016.10.015 27806833

[8] Sipola-Leppanen M Vaarasmaki M Tikanmaki M Hovi P Miettola S Ruokonen A. Cardiovascular risk factors in adolescents born preterm. Pediatrics. (2014) 134:e1072–81. 10.1542/peds.2013-4186 25180275

[9] Sipola-Leppanen M Vaarasmaki M Tikanmaki M Matinolli HM Miettola S Hovi P. Cardiometabolic risk factors in young adults who were born preterm. Am J Epidemiol. (2015) 181:861–73. 10.1093/aje/kwu443 25947956 PMC4445394

[10] Radtke JV. The paradox of breastfeeding-associated morbidity among late preterm infants. J Obstet Gynecol Neonatal Nurs. (2011) 40:9–24. 10.1111/j.1552-6909.2010.01211.x 21244492 PMC3216635

[11] Johnson S Evans TA Draper ES Field DJ Manktelow BN Marlow N. Neurodevelopmental outcomes following late and moderate prematurity: a population-based cohort study. Arch Dis Child Fetal Neonatal Ed. (2015) 100:F301–8. 10.1136/archdischild-2014-307684 25834170 PMC4484499

[12] Brockway M Benzies KM Carr E Aziz K. Breastfeeding self-efficacy and breastmilk feeding for moderate and late preterm infants in the family integrated care trial: a mixed methods protocol. Int Breastfeed J. (2018) 13:29. 10.1186/s13006-018-0168-7 29989087 PMC6035466

[13] Crippa BL Colombo L Morniroli D Consonni D Bettinelli ME Spreafico I. Do a few weeks matter? Late preterm infants and breastfeeding issues. Nutrients. (2019) 11:312. 10.3390/nu11020312 30717261 PMC6413139

[14] Huang P Zhou J Yin Y Jing W Luo B Wang J. Effects of breast-feeding compared with formula-feeding on preterm infant body composition: a systematic review and meta-analysis. Br J Nutr. (2016) 116:132–41. 10.1017/S0007114516001720 27181767

[15] Belfort MB Gillman MW Buka SL Casey PH McCormick MC. Preterm infant linear growth and adiposity gain: trade-offs for later weight status and intelligence quotient. J Pediatr. (2013) 163:1564–9 e2. 10.1016/j.jpeds.2013.06.032 23910982 PMC3834090

[16] Brumbaugh JE Conrad AL Lee JK DeVolder IJ Zimmerman MB Magnotta VA. Altered brain function, structure, and developmental trajectory in children born late preterm. Pediatr Res. (2016) 80:197–203. 10.1038/pr.2016.82 27064239 PMC4990473

[17] Lapillonne A O'Connor DL Wang D Rigo J. Nutritional recommendations for the late-preterm infant and the preterm infant after hospital discharge. J Pediatr. (2013) 162(Suppl.3):S90–100. 10.1016/j.jpeds.2012.11.058 23445854

[18] Blackwell MT Eichenwald EC McAlmon K Petit K Linton PT McCormick MC. Interneonatal intensive care unit variation in growth rates and feeding practices in healthy moderately premature infants. J Perinatol. (2005) 25:478–85. 10.1038/sj.jp.7211302 15889133

[19] Brown LD Hay WW. The nutritional dilemma for preterm infants: how to promote neurocognitive development and linear growth, but reduce the risk of obesity. J Pediatr. (2013) 163:1543–5. 10.1016/j.jpeds.2013.07.042 24018016

[20] Michalski MC Briard V Michel F Tasson F Poulain P. Size distribution of fat globules in human colostrum, breast milk, and infant formula. J Dairy Sci. (2005) 88:1927–40. 10.3168/jds.S0022-0302(05)72868-X 15905422

[21] Gallier S Gragson D Jimenez-Flores R Everett D. Using confocal laser scanning microscopy to probe the milk fat globule membrane and associated proteins. J Agric Food Chem. (2010) 58:4250–7. 10.1021/jf9032409 20218614 PMC2853928

[22] Lopez C Menard O. Human milk fat globules: polar lipid composition and in situ structural investigations revealing the heterogeneous distribution of proteins and the lateral segregation of sphingomyelin in the biological membrane. Colloids Surf B Biointerfaces. (2011) 83:29–41. 10.1016/j.colsurfb.2010.10.039 21126862

[23] Straarup EM Lauritzen L Faerk J Hoy Deceased CE Michaelsen KF. The stereospecific triacylglycerol structures and Fatty Acid profiles of human milk and infant formulas. J Pediatr Gastroenterol Nutr. (2006) 42:293–9. 10.1097/01.mpg.0000214155.51036.4f 16540799

[24] Innis SM Dyer R Nelson CM. Evidence that palmitic acid is absorbed as sn-2 monoacylglycerol from human milk by breast-fed infants. Lipids. (1994) 29:541–5. 10.1007/BF02536625 7990660

[25] Nowacki J Lee HC Lien R Cheng SW Li ST Yao M. Stool fatty acid soaps, stool consistency and gastrointestinal tolerance in term infants fed infant formulas containing high sn-2 palmitate with or without oligofructose: a double-blind, randomized clinical trial. Nutr J. (2014) 13:105. 10.1186/1475-2891-13-105 25373935 PMC4273321

[26] Yao M Lien EL Capeding MR Fitzgerald M Ramanujam K Yuhas R. Effects of term infant formulas containing high sn-2 palmitate with and without oligofructose on stool composition, stool characteristics, and bifidogenicity. J Pediatr Gastroenterol Nutr. (2014) 59:440–8. 10.1097/MPG.0000000000000443 24840511 PMC4222706

[27] Armand M Hamosh M Mehta NR Angelus PA Philpott JR Henderson TR. Effect of human milk or formula on gastric function and fat digestion in the premature infant. Pediatr Res. (1996) 40:429–37. 10.1203/00006450-199609000-00011 8865280

[28] Gallier S Vocking K Post JA Van De Heijning B Acton D Van Der Beek EM. A novel infant milk formula concept: mimicking the human milk fat globule structure. Colloids Surf B Biointerfaces. (2015) 136:329–39. 10.1016/j.colsurfb.2015.09.024 26432620

[29] Baumgartner S van de Heijning BJM Acton D Mensink RP. Infant milk fat droplet size and coating affect postprandial responses in healthy adult men: a proof-of-concept study. Eur J Clin Nutr. (2017) 71:1108–13. 10.1038/ejcn.2017.50 28422122

[30] Oosting A van Vlies N Kegler D Schipper L Abrahamse-Berkeveld M Ringler S. Effect of dietary lipid structure in early postnatal life on mouse adipose tissue development and function in adulthood. Br J Nutr. (2014) 111:215–26. 10.1017/S0007114513002201 23845308

[31] Baars A Oosting A Engels E Kegler D Kodde A Schipper L. Milk fat globule membrane coating of large lipid droplets in the diet of young mice prevents body fat accumulation in adulthood. Br J Nutr. (2016) 115:1930–7. 10.1017/S0007114516001082 27040581 PMC4863696

[32] Teller IC Hoyer-Kuhn H Bronneke H Nosthoff-Horstmann P Oosting A Lippach G. Complex lipid globules in early-life nutrition improve long-term metabolic phenotype in intra-uterine growth-restricted rats. Br J Nutr. (2018) 120:763–76. 10.1017/S0007114518001988 30109842

[33] Schipper L van Dijk G Broersen LM Loos M Bartke N Scheurink AJ. A postnatal diet containing phospholipids, processed to yield large, phospholipid-coated lipid droplets, affects specific cognitive behaviors in healthy male mice. J Nutr. (2016) 146:1155–61. 10.3945/jn.115.224998 27146919

[34] Breij LM Abrahamse-Berkeveld M Vandenplas Y Jespers SNJ de Mol AC Khoo PC. An infant formula with large, milk phospholipid-coated lipid droplets containing a mixture of dairy and vegetable lipids supports adequate growth and is well-tolerated in healthy, term infants. Am J Clin Nutr. (2019) 109:586–96. 10.1093/ajcn/nqy322 30793165 PMC6408203

[35] UNICEF. UNICEF Baby Friendly. Available online at: https://www.unicef.org.uk/babyfriendly/ (accessed March 31, 2020).

[36] National Health Service. Pregnancy and Baby. (2016). Available online at: https://www.nhs.uk/conditions/baby/weaning-and-feeding/vitamins-for-children/ (accessed March 31, 2020).

[37] Agostoni C Buonocore G Carnielli VP De Curtis M Darmaun D Decsi T. Enteral nutrient supply for preterm infants: commentary from the European Society of Paediatric Gastroenterology, Hepatology and Nutrition Committee on Nutrition. J Pediatr Gastroenterol Nutr. (2010) 50:85–91. 10.1097/MPG.0b013e3181adaee0 19881390

[38] Service of NSN RoSNISaRAUD. UK Data Service 2017. Available online at: 10.5257/census/aggregate-2011-2 (accessed March 31, 2020).

[39] Townsend PP. Health and Deprivation. Inequality and the North. London: Croom Helm Ltd. (1988).

[40] Organization WH. Child Growth: www.who.int. Available online at: https://www.who.int/childgrowth/en (accessed March 31, 2020).

[41] Mazicioglu MM Hatipoglu N Ozturk A Cicek B Ustunbas HB Kurtoglu S. Waist circumference and mid-upper arm circumference in evaluation of obesity in children aged between 6 and 17 years. J Clin Res Pediatr Endocrinol. (2010) 2:144–50. 10.4274/jcrpe.v2i4.144 21274313 PMC3005693

[42] Daly-Wolfe KM Jordan KC Slater H Beachy JC Moyer-Mileur LJ. Mid-arm circumference is a reliable method to estimate adiposity in preterm and term infants. Pediatr Res. (2015) 78:336–41. 10.1038/pr.2015.103 26020147

[43] Goswami I Rochow N Fusch G Liu K Marrin ML Heckmann M. Length normalized indices for fat mass and fat-free mass in preterm and term infants during the first six months of life. Nutrients. (2016) 8:417. 10.3390/nu8070417 27399768 PMC4963893

[44] Riley AW Trabulsi J Yao M Bevans KB DeRusso PA. Validation of a parent report questionnaire: the infant gastrointestinal symptom questionnaire. Clin Pediatr (Phila). (2015) 54:1167–74. 10.1177/0009922815574075 25758425 PMC4564761

[45] Llewellyn CH van Jaarsveld CH Johnson L Carnell S Wardle J. Development and factor structure of the baby eating behaviour questionnaire in the gemini birth cohort. Appetite. (2011) 57:388–96. 10.1016/j.appet.2011.05.324 21672566

[46] Mallan KM Daniels LA de Jersey SJ. Confirmatory factor analysis of the baby eating behaviour questionnaire and associations with infant weight, gender and feeding mode in an Australian sample. Appetite. (2014) 82:43–9. 10.1016/j.appet.2014.06.026 25009080

[47] Volger S Estorninos EM Capeding MR Lebumfacil J Radler DR Scott Parrott J. Health-related quality of life, temperament, and eating behavior among formula-fed infants in the Philippines: a pilot study. Health Qual Life Outcomes. (2018) 16:121. 10.1186/s12955-018-0944-5 29884187 PMC5994097

[48] Wardle J Guthrie CA Sanderson S Rapoport L. Development of the children's eating behaviour questionnaire. J Child Psychol Psychiatry. (2001) 42:963–70. 10.1111/1469-7610.00792 11693591

[49] Svensson V Lundborg L Cao Y Nowicka P Marcus C Sobko T. Obesity related eating behaviour patterns in Swedish preschool children and association with age, gender, relative weight and parental weight–factorial validation of the children's eating behaviour questionnaire. Int J Behav Nutr Phys Act. (2011) 8:134. 10.1186/1479-5868-8-134 22152012 PMC3286377

[50] Marriott LD Inskip HM Borland SE Godfrey KM Law CM Robinson SM. What do babies eat? Evaluation of a food frequency questionnaire to assess the diets of infants aged 12 months. Public Health Nutr. (2009) 12:967–72. 10.1017/S1368980008003388 18702837

[51] Marriott LD Robinson SM Poole J Borland SE Godfrey KM Law CM. What do babies eat? Evaluation of a food frequency questionnaire to assess the diets of infants aged 6 months. Public Health Nutr. (2008)11:751–6. 10.1017/S1368980007001292 18005490

[52] Blaggan S Guy A Boyle EM Spata E Manktelow BN Wolke D. A parent questionnaire for developmental screening in infants born late and moderately preterm. Pediatrics. (2014) 134:e55–62. 10.1542/peds.2014-0266 24982100

[53] Johnson S Wolke D Marlow N Preterm Infant Parenting Study G. Developmental assessment of preterm infants at 2 years: validity of parent reports. Dev Med Child Neurol. (2008) 50:58–62. 10.1111/j.1469-8749.2007.02010.x 18173632

[54] Martin AJ Darlow BA Salt A Hague W Sebastian L McNeill N. Performance of the Parent Report of Children's Abilities-Revised (PARCA-R) versus the bayley scales of infant development III. Arch Dis Child. (2013) 98:955–8. 10.1136/archdischild-2012-303288 24030249

[55] Stewart CJ Embleton ND Marrs ECL Smith DP Fofanova T Nelson A. Longitudinal development of the gut microbiome and metabolome in preterm neonates with late onset sepsis and healthy controls. Microbiome. (2017) 5:75. 10.1186/s40168-017-0295-1 28701177 PMC5508794

[56] Mai V Young CM Ukhanova M Wang X Sun Y Casella G. Fecal microbiota in premature infants prior to necrotizing enterocolitis. PLoS ONE. (2011) 6:e20647. 10.1371/journal.pone.0020647 21674011 PMC3108958

[57] Severance EG Yolken RH Eaton WW. Autoimmune diseases, gastrointestinal disorders and the microbiome in schizophrenia: more than a gut feeling. Schizophr Res. (2016) 176:23–35. 10.1016/j.schres.2014.06.027 25034760 PMC4294997

[58] Stewart CJ Marrs EC Magorrian S Nelson A Lanyon C Perry JD. The preterm gut microbiota: changes associated with necrotizing enterocolitis and infection. Acta Paediatr. (2012) 101:1121–7. 10.1111/j.1651-2227.2012.02801.x 22845166

[59] Skeath TS Stewart C Berrington JE. Decoding the bacterial microbiome of preterm babies—insights, unknowns and opportunities. Infant. (2014) 10:112−6. Available online at: https://www.infantjournal.co.uk/journal_article.html?id=6729

[60] Kalliomaki M Kirjavainen P Eerola E Kero P Salminen S Isolauri E. Distinct patterns of neonatal gut microflora in infants in whom atopy was and was not developing. J Allergy Clin Immunol. (2001) 107:129–34. 10.1067/mai.2001.111237 11150002

[61] Chernikova DA Madan JC Housman ML Zain-Ul-Abideen M Lundgren SN Morrison HG. The premature infant gut microbiome during the first 6 weeks of life differs based on gestational maturity at birth. Pediatr Res. (2018) 84:71–9. 10.1038/s41390-018-0022-z 29795209 PMC6082716

[62] Forsgren M Isolauri E Salminen S Rautava S. Late preterm birth has direct and indirect effects on infant gut microbiota development during the first six months of life. Acta Paediatr. (2017) 106:1103–9. 10.1111/apa.13837 28316118 PMC5763336

[63] Pediatrics AAo. Clinical Testing of Infant Formulas with Respect to Nutritional Suitability for Term Infants. Elk Grove Village: American Academy of Pediatrics, Committee on Nutrition (1988).

[64] Pediatrics AAo. Clinical Testing of Infant Formulas with Respect to Nutritional Suitability for Term Infants. Report to the FDA. Elk Grove Village, IL: American Academy of Pediatrics (1988).

[65] Santos IS Matijasevich A Domingues MR Barros AJ Victora CG Barros FC. Late preterm birth is a risk factor for growth faltering in early childhood: a cohort study. BMC Pediatr. (2009) 9:71. 10.1186/1471-2431-9-71 19917121 PMC2780991

[66] Sammallahti S Heinonen K Andersson S Lahti M Pirkola S Lahti J. Growth after late-preterm birth and adult cognitive, academic, and mental health outcomes. Pediatr Res. (2017) 81:767–74. 10.1038/pr.2016.276 28056012

[67] Cicek B Ozturk A Mazicioglu MM Kurtoglu S. Arm anthropometry indices in Turkish children and adolescents: changes over a three-year period. J Clin Res Pediatr Endocrinol. (2014) 6:216–26. 10.4274/jcrpe.1574 25541892 PMC4293656

[68] Gupta P Mital R Kumar B Yadav A Jain M Upadhyay A. Physical Growth, Morbidity Profile and Mortality Among Healthy Late Preterm Neonates. Indian Pediatr. (2017) 54:629–34. 10.1007/s13312-017-1123-1 28607209

[69] Ramel SE Gray HL Davern BA Demerath EW. Body composition at birth in preterm infants between 30 and 36 weeks gestation. Pediatr Obes. (2015) 10:45–51. 10.1111/j.2047-6310.2013.00215.x 24470220

[70] Villar J Giuliani F Bhutta ZA Bertino E Ohuma EO Ismail LC. Postnatal growth standards for preterm infants: the Preterm Postnatal Follow-up Study of the INTERGROWTH-21(st) Project. Lancet Glob Health. (2015) 3:e681–91. 10.1016/S2214-109X(15)00163-1 26475015

